# PARP Inhibition Restores Extrinsic Apoptotic Sensitivity in Glioblastoma

**DOI:** 10.1371/journal.pone.0114583

**Published:** 2014-12-22

**Authors:** Georg Karpel-Massler, Fresia Pareja, Pascaline Aimé, Chang Shu, Lily Chau, Mike-Andrew Westhoff, Marc-Eric Halatsch, John F. Crary, Peter Canoll, Markus D. Siegelin

**Affiliations:** 1 Department of Pathology & Cell Biology, Columbia University Medical Center, New York, New York, United States of America; 2 Department of Pediatrics and Adolescent Medicine, Ulm University Medical Center, Ulm, Germany; 3 Department of Neurosurgery, Ulm University Medical Center, Ulm, Germany; University of Navarra, Spain

## Abstract

**Background:**

Resistance to apoptosis is a paramount issue in the treatment of Glioblastoma (GBM). We show that targeting PARP by the small molecule inhibitors, Olaparib (AZD-2281) or PJ34, reduces proliferation and lowers the apoptotic threshold of GBM cells *in vitro* and *in vivo*.

**Methods:**

The sensitizing effects of PARP inhibition on TRAIL-mediated apoptosis and potential toxicity were analyzed using viability assays and flow cytometry in established GBM cell lines, low-passage neurospheres and astrocytes *in vitro*. Molecular analyses included western blots and gene silencing. *In vivo*, effects on tumor growth were examined in a murine subcutaneous xenograft model.

**Results:**

The combination treatment of PARP inhibitors and TRAIL led to an increased cell death with activation of caspases and inhibition of formation of neurospheres when compared to single-agent treatment. Mechanistically, pharmacological PARP inhibition elicited a nuclear stress response with up-regulation of down-stream DNA-stress response proteins, e.g., CCAAT enhancer binding protein (C/EBP) homology protein (CHOP). Furthermore, Olaparib and PJ34 increased protein levels of DR5 in a concentration and time-dependent manner. In turn, siRNA-mediated suppression of DR5 mitigated the effects of TRAIL/PARP inhibitor-mediated apoptosis. In addition, suppression of PARP-1 levels enhanced TRAIL-mediated apoptosis in malignant glioma cells. Treatment of human astrocytes with the combination of TRAIL/PARP inhibitors did not cause toxicity. Finally, the combination treatment of TRAIL and PJ34 significantly reduced tumor growth *in vivo* when compared to treatment with each agent alone.

**Conclusions:**

PARP inhibition represents a promising avenue to overcome apoptotic resistance in GBM.

## Introduction

Certain cancers display a highly treatment resistant phenotype. A prototype of these tumors represents Glioblastoma (GBM), which despite vast treatment efforts carries a grim prognosis as reflected by a median overall survival of less than 15 months [1]. One mechanism by which GBM can evade therapy is resistance to apoptotic cell death. Restoring apoptotic sensitivity is therefore of paramount importance to render GBMs sensitive to drug therapy. One way to make treatment resistant cancers amenable to drug treatment is the administration of combinatorial drug regimens. Such treatments may overcome primary and acquired resistance to therapy. Virtually all GBMs develop secondary treatment resistance after administration of either Temozolomide (TMZ), radiation or the combination of TMZ + radiation. Since the DNA repair enzyme poly(ADP-ribose) polymerase (PARP) is expressed at higher levels in tumor cells when compared to benign tissues and cells [2], [3], PARP may therefore represent a tumor specific treatment target. Moreover, while assisting rapid dividing cancer cells with DNA-repair, PARP counteracts apoptotic cell death. Consistent with this idea, interference with PARP by RNA silencing or PARP inhibitors render cancer cells more prone to the cytotoxic effects of DNA-damage inducing treatment modalities, such as radiation, topoisomerase inhibitors or alkylating reagents (i.e. Temozolomide) [4], [5]. We focus on the PARP inhibitor, Olaparib (Olap, AZD-2281), which penetrates the blood-brain barrier and has already reached clinical trials in GBM patients. Our data demonstrate that Olaparib overcomes apoptotic resistance and sensitizes GBM cells for death receptor-mediated apoptosis induced by TRAIL (Tumor necrosis factor-related apoptosis-inducing ligand) through up-regulation of TRAIL receptor 2 (DR5) independent of their *TP53* status. In addition, PARP-1 specific siRNA, as well as PJ34 [6], another pharmacological PARP inhibitor, also enhanced extrinsic apoptosis in GBM cells *in vitro* and *in vivo*. Since TRAIL is known for its tumor specificity, the combination treatment of PARP inhibitors with TRAIL may be an ideal drug combination therapy with potential little side effects.

## Material and Methods

### Ethics statement

All procedures were in accordance with Animal Welfare Regulations and approved by IACUC Columbia University Medical Center. The study was reviewed and approved by the institutional review board at Columbia University Medical Center. Human tissue samples were anonymized prior to access by the researchers.

### Cell lines and reagents

Human GBM cell lines, LN229 (p53 mutated), U87 (p53 wild-type), T98G (p53 mutated) and U373 (p53 mutated) were purchased from the American Type Culture Collection (Manassas, VA) and were cultured as previously described [7]. The U87-EGFRvIII cells were a kind gift from Dr. Frank Furnari (Ludwig Institute for Cancer Research, La Jolla, CA). The human astrocytes were purchased from Sciencell Research Laboratories (Carlsbad, CA). MDA-MB-436 and MDA-MB-468 are breast cancer cell lines and were purchased from the American Type Culture Collection, Manassas, VA. Olaparib (Olap) and recombinant human TRAIL (rhTRAIL) were purchased from LC Laboratories (Woburn, MA) and Peprotech (Rocky Hill, NJ), respectively. The PARP-inhibitor, PJ34, was purchased from Selleckchem (Houston, TX). The low passage neurosphere GS9-6 stem-like glioma cell culture [8] was cultured as previously described [9], [10]. Cells were either treated with TRAIL, PJ34, Olaparib or in combination with TRAIL + PJ34 or TRAIL + Olaparib.

### Antibodies and Western Blotting

Antibodies to CHOP (1∶500, CST: Cell Signaling Technology, Danvers, MA), DR5 (1∶500; CST), cleaved caspase-3 (1∶250; CST), caspase-8 (total form), cleaved caspase-8 (1∶250; CST), XIAP (1∶500, CST), Survivin (1∶500; CST), Bcl2 (1∶500; CST), c-FLIP (1∶500; CST), p-p53 (serine 15) (1∶500, CST), p-CHk1 (Serine 345) (1∶500; CST), p-H2AX (Serine 15), DR4 (1∶500, Abcam) Actin (1∶2000, Sigma Aldrich), BAX (1∶500; CST), PARP-1 (1∶1000; CST) and cleaved PARP (Asp214, 1∶1000; CST) were used. Western Blotting was performed as described previously [11].

### Analysis of cellular viability, apoptosis and cell cycle

3(4,5-dimethyl-thyazoyl-2-yl)2,5 diphenyltetrazolium bromide (MTT) colorimetric assays were conducted as previously described [12]. For apoptosis determination, cells were stained and analyzed with Annexin V (FITC)/Propidium iodide as previously described [12]. For cell cycle analysis, cells were harvested and fixed in ethanol. After fixation overnight, cells were stained with Propidium iodide staining solution (# 4087, Cell Signaling Technology). For analysis of loss of mitochondrial membrane potential cells were stained with JC-1 and analyzed by flow cytometry as described [11].

### Transfections of siRNAs

Non-targeting siRNA-pool (ON-TARGETplus Non-targeting Pool, # D-001810-10-05) and siRNA against DR5 (SMARTpool: ON-TARGETplus TNFRSF10B siRNA, L-004448-00-0005), PARP-1 (SMARTpool: ON-TARGETplus PARP1 siRNA, L-006656-03-0005), BAX (SMARTpool: ON-TARGETplus BAX siRNA, L-003308-01-0005) and CASP8 (SMARTpool: ON-TARGETplus CASP8 siRNA, L-003466-00-0005) were purchased from Thermo Fisher Scientific (Pittsburgh, PA) and transfected as previously described [12].

### Neurosphere formation assay

GS9-6 cells were mechanically dissociated and plated at a density of 500 cells per well in 12 well plates. Twenty-four hours later, cells were treated with TRAIL and Olaparib, singly or in combination. Following 10 days, neurosphere formation was assessed by counting the number of neurospheres that harbor at least 25 cells per sphere as described in [13], [14]. Experiments were performed at least in duplicates and statistical analysis was performed.

### Subcutaneous xenografts

U87 cells were grown as subcutaneous tumors in 6-8 week old *SCID SHO* mice. To establish the tumors and the respective treatment groups, U87 cells were pretreated with DMSO, TRAIL (100 ng/ml), PJ34 (40 µM) or the combination of both reagents for 2 hours to form 4 different treatment groups. For each treatment condition/group 3 million viable cells for the establishment of each tumor were injected subcutaneously. After injection, animals were monitored daily for the appearance of tumors. Tumors were measured with a caliper and sizes calculated according to the standard formula: (length * width^2^)*0.5. Once tumors reached a size of more than 1 cm^3^ animals were euthanized. All procedures were in accordance with Animal Welfare Regulations and approved by Columbia IACUC.

### Statistical analysis

Data were analyzed by two-sided unpaired t–tests, using GraphPad Prism software, or one-way analysis of variance followed by Tukey's Multiple Comparison Test. Values are provided as mean ± SD or mean ± SEM of replicates of a representative experiment out of at least 2 independent determinations. A p value of less than 0.05 (p<0.05) was accepted as statistically significant.

## Results

### PARP-1 displays a heterogeneous expression pattern in GBM tissue specimens, GBM cell lines and GBM neurosphere cell cultures

To determine if PARP-1 is a suitable target for the treatment of malignant glioma we assessed the expression levels in GBM cells and 34 GBM tissue specimens. All GBM tissue specimens demonstrated detectable PARP staining, which had a predominantly nuclear localization with some faint staining in the cytoplasm (Figure A in S1 Fig.). About 68% of the tumors revealed moderate expression, whereas 32% showed strong expression (S1 Table). The staining intensity was heterogeneous among the different tumors as well as within a specific tumor. Normal brain tissue showed less PARP staining (Figure A in S1 Fig.). Residing glial cells demonstrated detectable PARP-1 expression. Neurons showed cytoplasmic and nuclear staining, which was mostly confined to the nucleolus. Next, the protein expression levels of PARP-1 were determined being lowest in U87 and higher in neurosphere cultures with the exception of GS9-6, which showed lower protein expression levels of PARP-1 compared to NCH644 and NCH690, respectively (Figure B in S1 Fig.).

### Inhibition of PARP-1 by Olaparib decreases proliferation of GBM cells

We tested whether the PARP-1 inhibitor Olaparib (Figure C in S1 Fig.) is capable of apoptosis induction by itself. LN229 (higher levels of PARP-1) and U87 (lower levels of PARP-1) cells were treated with increasing concentrations of Olaparib. Olaparib elicited a minimal increase in apoptosis in LN229 cells 72 h after treatment (Figure D in S1 Fig.). However, Olaparib had a significant effect on the cell cycle progression, demonstrating a G2/M arrest in LN229 cells (Figure D in S1 Fig.). In contrast, there was little induction of apoptosis as indicated by a low proportion of cells in the sub-G1 fraction. We also treated LN229 and U87 cells with increasing concentrations of Olaparib, resulting in a dose-dependent inhibition of proliferation which was more accentuated in LN229 cells (Figure E in S1 Fig.), consistent with their higher expression of PARP-1 protein. In addition, U87-EGFRvIII as well as the stem cell-like neurosphere culture, GS9-6, were treated with increasing concentrations of Olaparib and revealed a moderate loss in cellular viability (Figure E in S1 Fig.).

### The combination of Olaparib and TRAIL cooperates to induce loss of cellular viability in GBM cells and triple-negative breast cancer cells

**Figure 1:**
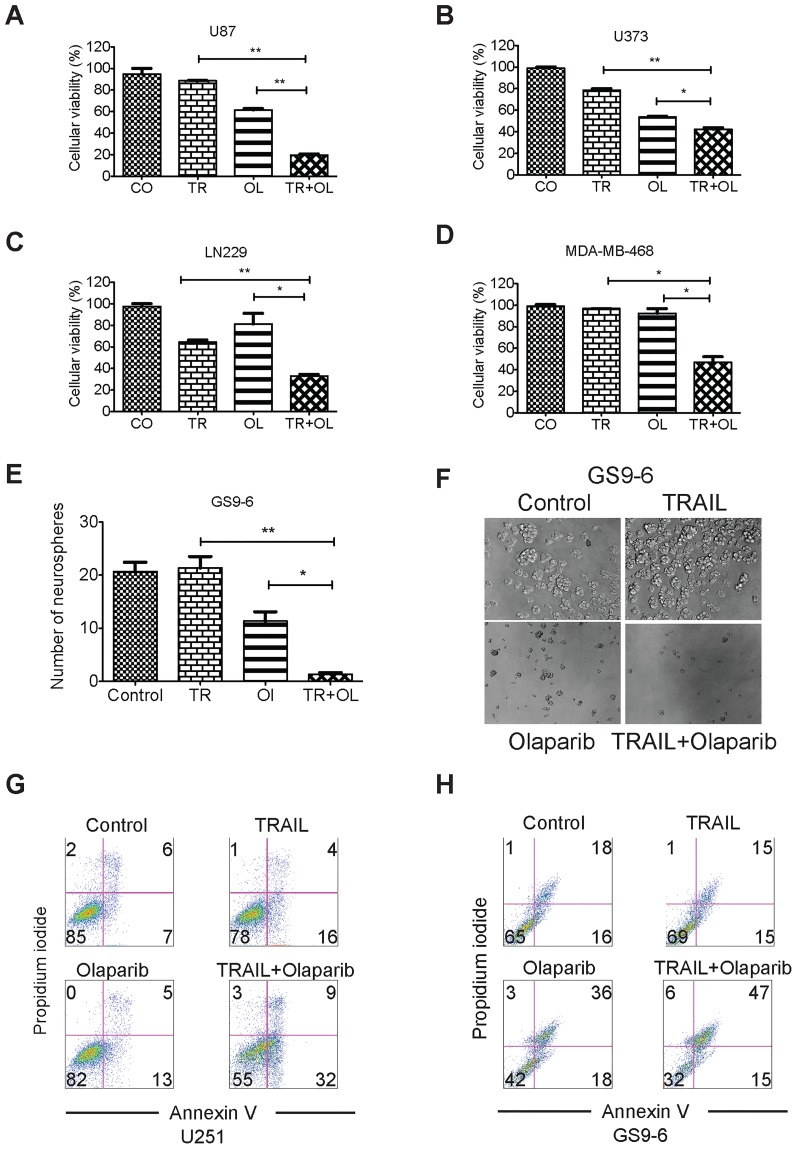
Cooperative cell death induction of the combination treatment with TRAIL and Olaparib. A–D): U87 (A), U373 (B), LN229 (C) and MDA-MB-468 (D) cells were treated with suboptimal dosages of TRAIL (A: 100 ng/ml, B: 25 ng/ml, C: TRAIL 200 ng/ml, D: 10 ng/ml), Olaparib (A–C: 10 µM, D: 5 µM) or the combination of both reagents for 48 hours. Thereafter, MTT assays were performed to determine cellular viability. E–F) GBM neurosphere culture (GS9-6) was treated with suboptimal dosages of TRAIL, Olaparib or the combination of both reagents and assessed for neurosphere formation 2 weeks after plating. G–H): U251 and GS9-6 (GBM neurosphere culture) cells were treated with suboptimal dosages of TRAIL, Olaparib or the combination of both reagents and stained with Annexin-V (FITC-conjugated) and Propidium iodide 24 hours after treatment. Cells were analyzed by flow cytometry to determine the fraction of apoptotic cells. Values are given as mean ± SEM of representative experiments. The unpaired t-test was used to calculate the p-values. A p-value of less than 0.05 (0.01 to 0.05) is indicated by one star “*”, whereas a p-value of less than 0.01 (0.001 to 0.01) is highlighted by two stars “**”. A p-value less than 0.001 is indicated by a star triplet (***). CO – Control, TR – TRAIL, OL – Olaparib, TR+OL – TRAIL + Olaparib.

To determine if Olaparib is capable of overcoming apoptotic resistance several established cell lines with different genetic backgrounds were treated with TRAIL, Olaparib or the combination of both drugs. Suboptimal dosages of TRAIL had mild to moderate effects on cellular viability in U87 (88.46%±0.2928), U373 (53.58%±0.7463) and LN229 GBM cells (81.33%±9.783) (Fig. 1A–C). Olaparib on its own also elicited mild to moderate effects on cellular viability in U87 (61.56%±1.279), U373 (53.58%±0.7463) and LN229 (81.33±9.783) GBM cells (Fig. 1A–C). However, the combination of both compounds caused a greater reduction of cellular viability in U87 (19.58%±1.094), U373 (42.29%±1.493) and LN229 (33.19%±1.475) GBM cell lines (Fig. 1A–C). In all three GBM cell lines the combination therapy resulted in a statistically significant (p<0.05) decrease in cellular viability when compared to the single agent treatments. It is noteworthy that the combination treatment does not require the presence of a functional p53 protein since U373 and LN229 harbor a mutated form of p53. To show that the favorable effect of the drug combination of TRAIL/Olaparib is not restricted to GBM we treated the triple-negative breast cancer cell line MDA-MB-468. This cell line lacks the expression of estrogen, progesterone and HER2 receptors and therefore is a model system of another current treatment challenge in oncology. MDA-MB-468 showed a minor response to either TRAIL (96.80%±0.05955) or Olaparib (92.31%±4.426), whereas the combination of both reagents (4.79%±5.393) induced a decrease in cellular viability in a synergistic manner (Fig. 1D).

### The combination of Olaparib and TRAIL is effective in low-passage *ex vivo* GBM cultures

Stem cell-like glioma cells are known to be responsible for the rapid recurrence of GBM and for their resistance to therapy. We tested if the combination treatment of TRAIL and Olaparib affects stem cell-like glioma cells and if neurosphere formation is impaired by the combination treatment. While TRAIL and Olaparib used as individual agents exerted minor effects on neurosphere formation, the combination of the two drugs significantly impaired the formation of neurospheres (Fig. 1E–F), suggesting that this combination treatment may affect the glioma stem cell-like fraction.

### The combination of Olaparib and TRAIL causes enhanced apoptotic cell death with enhanced activation of initiator and effector-caspases

To test the hypothesis that the combination of TRAIL/Olaparib enhances apoptosis, U251 GBM cells were treated with vehicle, TRAIL, Olaparib or the combination of both and stained with Annexin V and Propidium iodide prior to analysis by flow cytometry, which showed enhanced apoptosis in the combination treatment when compared to the single agent treatments (Fig. 1G and Figure A in S4 Fig.). Next, we determined if stem cell-like glioma cells that are known to be vigorously resistant to extrinsic apoptosis could be sensitized to TRAIL-mediated apoptosis. For this purpose, the primary neurosphere culture, GS9-6, was treated with vehicle, TRAIL, Olaparib and the combination of both for 24 hours. Similarly to the established GBM cells, the combination of Olaparib and TRAIL led to a significant increase in apoptosis induction as compared to the single agent treatments (Fig. 1H and Figure B in S4 Fig.), suggesting that the combined treatment of Olaparib with recombinant human TRAIL not only affects the bulk of the tumor cells but more importantly targets the stem cell population of GBM cells, which according to the recent literature may be responsible for the rapid recurrence of these tumors.

**Figure 2:**
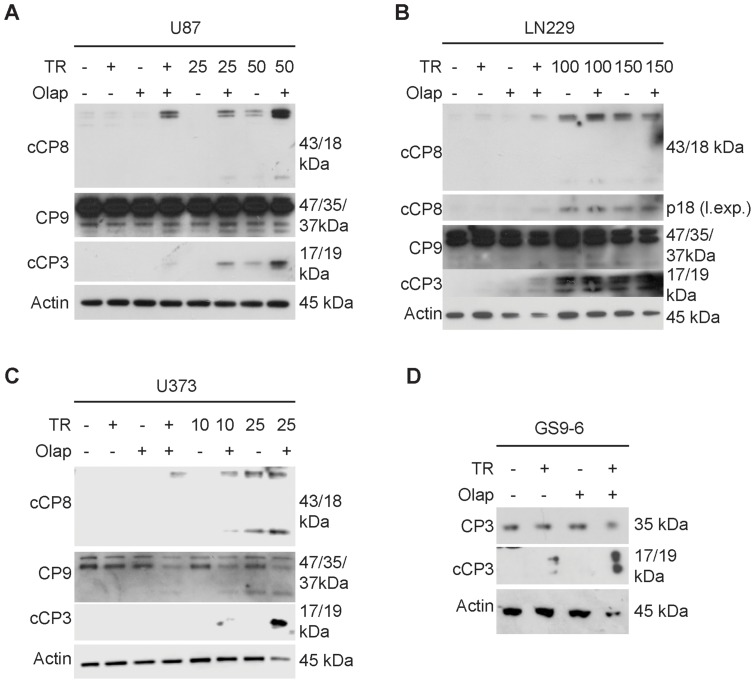
The combination therapy, consisting of TRAIL and Olaparib, elicits enhanced activation of initiator- and effector caspases. A–C) U87 (A), LN229 (B) and U373 (C) GBM cells were treated with TRAIL (ng/ml), Olaparib (10 µM) or the combination of both for 7 hours, subsequently harvested for immunoblotting and analyzed for the expression of cleaved caspase-8 (cCP8), full length caspase-9 (CP9) or effector caspase-3 (cCP3). In B the small fragment of cleaved caspase-8 is exposed longer (separate longer exposure (l. exp.)). Actin serves as a loading control. D) GBM neurosphere culture cells, GS9-6, were incubated with TRAIL, Olaparib or the combination of both reagents for 7 hours, subjected to immunoblotting and analyzed for full length caspase-3 (CP3) and cleaved caspase-3 (cCP3). Actin serves as a loading control. TR – TRAIL, Olap – Olaparib.

To elucidate the mechanism by which TRAIL/Olaparib elicit their effects on cellular viability, we hypothesized that it might involve enhanced activation of the apoptotic machinery. To test this, we conducted Western Blot analysis for activation (cleavage) of caspases in U87, U373, LN229 GBM cells and GS9-6 stem cell-like glioma cells in response to increasing dosages of TRAIL and Olaparib or the combination of both (Fig. 2A–D). We found that in all cell lines tested the combination treatment of TRAIL and Olaparib led to an enhanced activation of initiator- (caspase-8/9) and effector caspase-3 (Fig. 2A–D).

### Olaparib elicits an up-regulation of TRAIL receptor 2 (DR5) accompanied by an increase in CCAAT enhancer binding protein (C/EBP) homology protein (CHOP)/GADD153 expression in GBM cells

**Figure 3:**
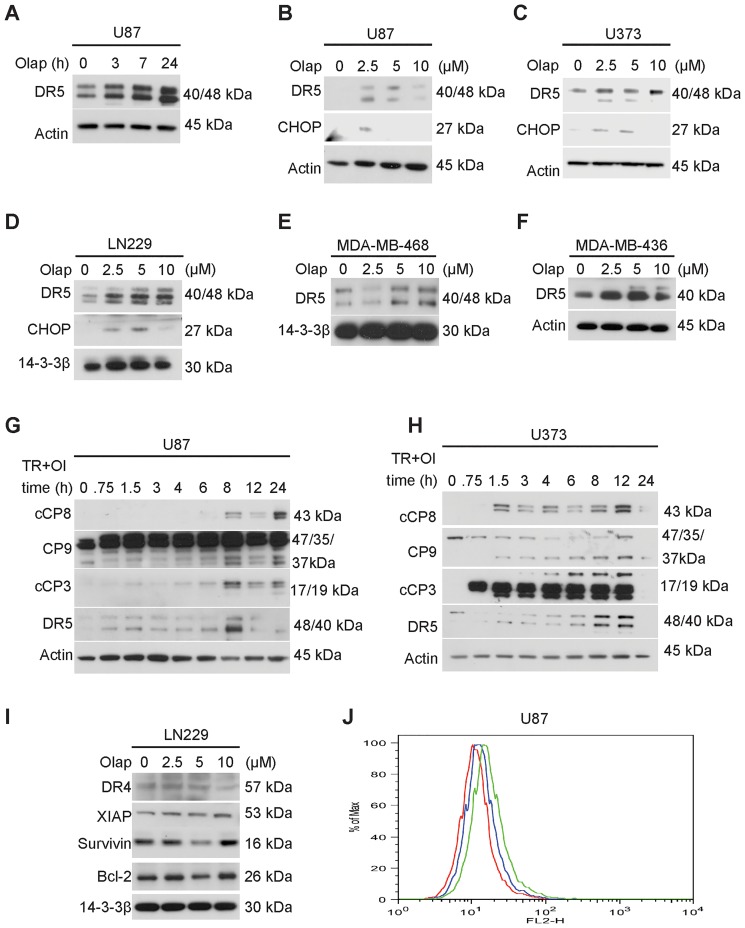
Olaparib elicits an increase in TRAIL receptor 2 (DR5) expression in GBM cells. A) U87 GBM cells were treated with Olaparib (10 µM) for the indicated time points, subjected to immunoblotting and analyzed for the expression of DR5. B–D) U87 (B), U373 (C) and LN229 GBM cells (D) were treated with increasing concentrations of Olaparib (µM) for 7 hours, subjected to immunoblotting and analyzed for the expression of CHOP and DR5. E–F) MDA-MB-468 (E) and MDA-MB-436 (F) were treated with increasing concentrations (µM) of Olaparib for 7 hours, harvested for immunoblotting and analyzed for the expression of DR5. G–H) U87 (G) and U373 (H) GBM cells were treated with the combination of TRAIL/Olaparib (U87: TRAIL 100 ng/ml, Olaparib 10 µM; U373: TRAIL 25 ng/ml Olaparib 10 µM) for a time course analysis, subjected to immunoblotting and analyzed for expression of cleaved caspase-8 (cCP8), full length caspase-9 (CP9), cCP3 (cleaved caspase-3) and DR5. Actin serves as a loading control. I) LN229 GBM cells were treated with increasing concentrations of Olaparib (µM), subjected to immunoblotting and analyzed for the expression of DR4, XIAP, Survivin and Bcl-2. J) U87 glioma cells were treated with Olaparib (5 µM) overnight, stained with a primary PE labeled antibody against DR5 and subjected to flow cytometry. A representative isotype control served as a control. The isotype-control is indicated in red, DR5 expression upon treatment with solvent is indicated in blue and DR5 expression after treatment with Olaparib is indicated in green. Olap – Olaparib, TR+OL – TRAIL + Olaparib.

Under the assumption that Olaparib may cause a cellular stress response we hypothesized that Olaparib may up-regulate TRAIL receptor 2 (DR5), which is a bona-fide example of a protein that is known to be downstream of various stress responses, including endoplasmic reticulum stress and nuclear stress [15]. To confirm this hypothesis, U87 GBM cells were treated with Olaparib and a time course analysis of the expression of DR5 was conducted by Western Blotting. As early as three hours after treatment, an increase in expression of death receptor 5 (DR5) was appreciated, increasing further at 7 hours and culminating at 24 hours (Fig. 3A). Next, we studied the expression level of DR5 after treatment with increasing concentrations of Olaparib at 7 hours (Fig. 3B–D). U87, LN229 and U373 GBM cells revealed the strongest induction of DR5 between 5–10 µM Olaparib (Fig. 3B–D). In addition, we also confirmed that triple-negative breast cancer cells (MDA-MB-468, MDA-MB-436) revealed an increase in DR5 expression after Olaparib treatment (Fig. 3E and 3F), suggesting that the mechanism of DR5 up-regulation is not only applicable to GBM but also to other tumor entities. Furthermore, it is known that the stress response transcription factor CCAAT enhancer binding protein (C/EBP) homology protein (CHOP) is often involved in drug-mediated DR5 increase [15]. Therefore, we tested as to whether CHOP is upregulated after increasing concentrations of Olaparib. Olaparib caused a concentration-dependent increase of CHOP in U87, LN229 and U373 GBM cells that paralleled the increase of DR5 levels (Fig. 3B–D), indicating that CHOP may be involved in DR5 modulation after Olaparib treatment.

### Highest levels of DR5 coincided with the strongest activation of initiator and effector caspases in the combination treatment of Olaparib and TRAIL

A time course analysis of cells treated with the combination of TRAIL/Olaparib was conducted to explore whether the up-regulation of DR5 was associated with an increase in cleavage/activation of initiator/effector caspases. To this end, U87 and U373 GBM cells were exposed to the combination of TRAIL/Olaparib and subsequently analyzed for cleavage of caspase-8/-9/-3 and expression of DR5 (Fig. 3G–H). We provided evidence that the TRAIL-resistant U87 and U373 cells revealed the strongest induction of DR5 along with the most pronounced activation of caspases (Fig. 3G–H), supporting the hypothesis that DR5 is an instrumental factor for TRAIL/Olaparib-mediated cell death in high-grade gliomas. To further elucidate as to why Olaparib lowers the apoptotic threshold in GBM cells we analyzed the expression of pivotal key molecules that confer resistance to apoptosis. While XIAP did not change significantly, Bcl-2 and Survivin protein expression were mildly affected by Olaparib at 5 µM (Fig. 3I). At 10 µM of Olaparib no significant change was evident. XIAP and DR4 did not change significantly (Fig. 3I). These results reinforce the notion that DR5 is the key modulator of TRAIL/Olaparib-mediated apoptosis in this setting.

### Olaparib increases membranous DR5 expression in GBM cells

Following their processing, death receptors are integrated into the plasma membrane, where they can interact with death ligands. Thus, we aimed to determine whether Olaparib also elevates the expression of DR5 in the plasma membrane (Fig. 3J). We observed that treatment with Olaparib induced an increase in the levels of DR5 (green), compared to DMSO treated cells (blue) and the respective antibody isotype control (red) (Fig. 3J).

### Olaparib elicits a nuclear stress response with up-regulation of CHOP in a time-dependent manner in GBM cells, and siRNA-mediated suppression of CHOP attenuates TRAIL/Olaparib-mediated increase of DR5

**Figure 4:**
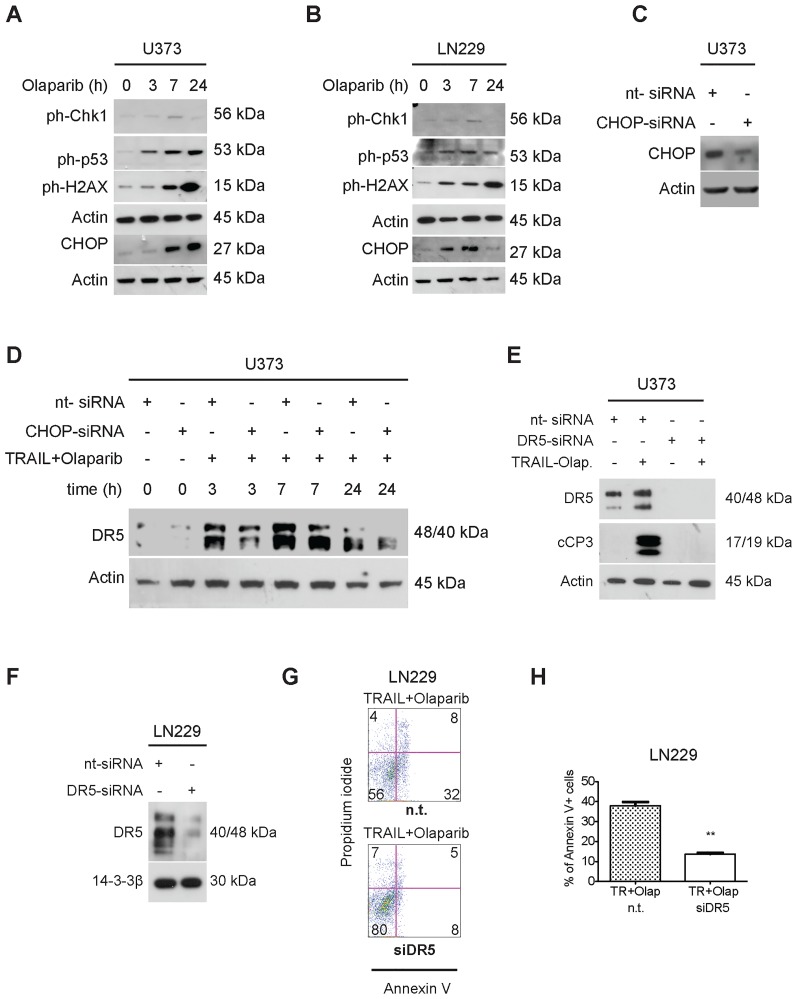
Olaparib causes a nuclear stress response in a time-dependent manner in GBM cells and regulates DR5 expression through CHOP. A–B) U373 (A) and LN229 (B) were treated with Olaparib (10 µM) for 3, 7, and 24 h. Cells were harvested for immunoblotting at the indicated timepoints and subjected to analysis for expression of ph-Chk1 (Serine 345), ph-p53 (Serine 15), ph-H2AX (Serine 139) and CHOP. C) U373 GBM cells were transfected with a non-targeting or a CHOP specific siRNA. 72 hours later, cells were harvested, subjected to immunoblotting and analyzed for CHOP expression. D) U373 GBM cells were transfected as indicated with either a non-targeting or a CHOP-specific siRNA. 72 hours later cells were treated with TRAIL/Olaparib and harvested for immunoblotting at the indicated time points. Thereafter, protein expression for DR5 was determined by immunoblotting. E) U373 GBM cells were transfected with a non-targeting or a DR5-specific siRNA. 72 hours after transfection cells were treated with the combination of TRAIL (50 ng/ml) and Olaparib (10 µM) for 7 hours, harvested for immunoblotting and analyzed for the expression of DR5 and cCP3. F) LN229 glioma cells were transfected with a non-targeting siRNA or a DR5-specific siRNA. 72 hours after transfection, cells were harvested for immunoblotting and DR5 expression was determined. G–H) LN229 cells transfected with a non-targeting (n.t.) or a DR5-specific siRNA were treated with TRAIL (200 ng/ml) and Olaparib (10 µM), stained with Annexin V/Propidium iodide and analyzed by flow cytometry. A p-value of less than 0.01 is indicated by two stars “**”. Columns, mean; bars, SEM. TR -TRAIL, Olap – Olaparib.

To determine the mechanistic properties of Olaparib we conducted a time course analysis for the appearance/up-regulation of molecules related to nuclear stress in U373 and LN229 GBM cells (Fig. 4A–B). Olaparib caused a cellular stress response in GBM cells, resulting in an up-regulation of CHOP, ph-Chk1, ph-p53 (Ser15) and ph-H2AX (Ser139) (Fig. 4A–B). Depending on the cell line, evidence for a nuclear stress response was observed as early as three hours after treatment with Olaparib (Fig. 4A–B). The presence of an early nuclear stress response with an up-regulation of CHOP elicited by Olaparib suggested that Olaparib may modulate the expression of downstream factors related to the cellular stress response, such as DR5 which is upregulated after Olaparib treatment, see Fig. 3. CHOP up-regulation also paralleled the increase of DR5 (Fig. 3B–D). Therefore, we determined if silencing of CHOP may inhibit the Olaparib/TRAIL-mediated increase in DR5 protein levels. We employed a siRNA that specifically suppressed the expression of CHOP. U373 GBM cells were either transfected with a non-targeting siRNA or a CHOP-specific siRNA (Fig. 4C) and subsequently treated with the combination of TRAIL and Olaparib. Cells transfected with CHOP-specific siRNA oligonucleotides showed a decrease in the up-regulation of DR5 after treatment with TRAIL/Olaparib at 3, 7 and 24 hours (Fig. 4D). These results support the hypothesis that CHOP is implicated in the increase in DR5 protein levels mediated by TRAIL/Olaparib.

### Specific suppression of DR5 by siRNA mitigates TRAIL/Olaparib-mediated apoptosis and activation of effector caspase-3

To test the hypothesis that DR5 is in fact a key molecule in TRAIL/Olaparib-mediated cell death GBM cell lines were transfected with either non-targeting siRNA or DR5-specific siRNA. U373 and U87 cells that were transfected with DR5-specific siRNA revealed suppression of DR5 protein levels when compared to the non-targeting transfected controls (Fig. 4E and Figure F in S2 Fig.). In addition, TRAIL/Olaparib-mediated increase in DR5 protein levels was potently attenuated by the DR5-specific siRNA (Fig. 4E). Furthermore, LN229 GBM cells transfected with DR5-specific siRNA were protected from TRAIL/Olaparib-mediated cell death, corroborating the importance of DR5 in TRAIL/Olaparib-mediated apoptosis (Fig. 4F–H). In addition, we observed that the proapoptotic effect of the combination therapy of TRAIL and PARP inhibitors is dependent on caspase-8 since silencing of this enzyme interferes with cell death induction (Figure A–E in S2 Fig.).

### PARP-inhibition by PJ34 as well as specific suppression of PARP-1 overcomes TRAIL resistance in GBM cells

**Figure 5:**
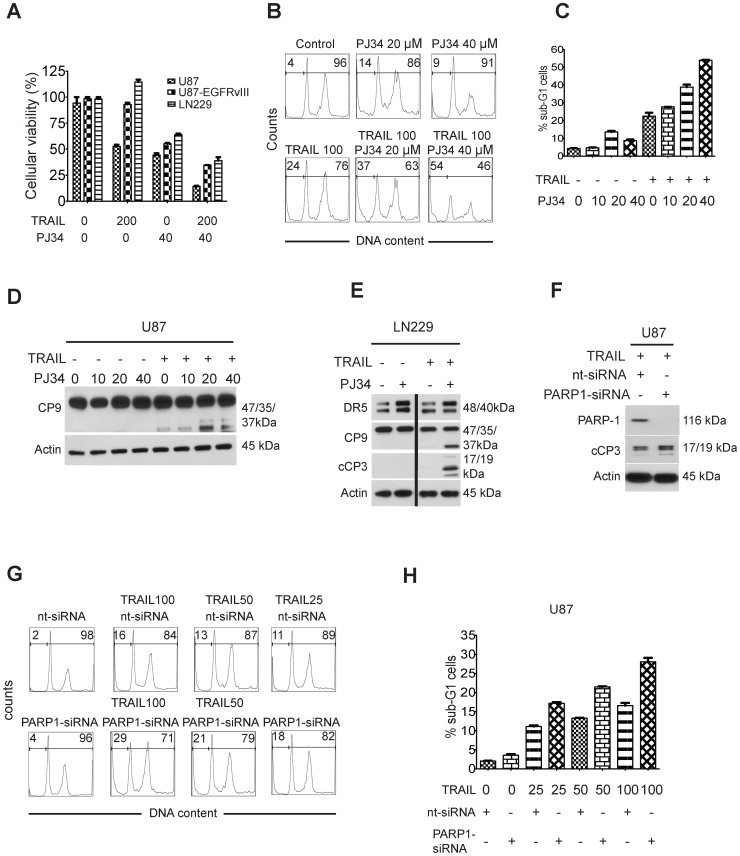
Inhibition of PARP-1 by the pharmacological inhibitor, PJ34, as well as PARP-1-specific siRNA-mediated suppression enhances TRAIL-mediated apoptosis in GBM cells. A) U87, U87 EGFRvIII and LN229 were treated with TRAIL (200 ng/ml), PJ34 (40 µM) or the combination of both for 72 hours. Subsequently, cells were analyzed by MTT-assay. Values are given as mean ± SEM. B,C) U87 GBM cells were treated with TRAIL, PJ34 or the combination of both for 24 hours and subsequently analyzed for specific apoptosis (sub-G1 fraction) by flow cytometry. In B, representative plots of these experiments are provided and C shows a quantitative analysis for these experiments. D) U87 GBM cells were treated with PJ34 (µM), TRAIL (100 ng/ml) or the combination of both with the indicated concentrations for 7 hours and subjected to immunoblotting analysis for cleavage of caspase-9. E) LN229 GBM cells were treated with PJ34 (20 µM), TRAIL (200 ng/ml) or the combination of both and analyzed for the expression of DR5, caspase-9 (CP9) and cleaved caspase-3 (cCP3). The vertical line on the immunoblot indicates that the first and second samples were noncontiguous, but run on the same gel simultaneously with the other samples. F) U87 gliobastoma cells were transfected with non-targeting or PARP-1-specific siRNA for 72 hours and subsequently incubated with TRAIL (100 ng/ml). Protein expression of cCP3 and PARP-1 was evaluated by immunoblotting. G–H) U87 cells were transfected with either non-targeting siRNA or with a siRNA specific for PARP-1. 72 hours after transfection cells were incubated with increasing concentrations of TRAIL (concentrations in ng/ml) and subsequently analyzed for apoptosis by flow cytometry (specific apoptosis, sub-G1 fraction). Shown are both representative plots (G) as well as a quantitation of the indicated results (H). Columns, mean; bars, SEM.

To determine whether the sensitizing effect of Olaparib to TRAIL-mediated apoptosis is restricted to this PARP inhibitor, we extended our analysis to another PARP-inhibitor, PJ34, and also analyzed the effects of specific siRNA-mediated suppression of PARP-1 on TRAIL-mediated apoptosis in GBM cells. U87, U87-EGFRvIII and LN229 GBM cells were treated with TRAIL, PJ34 or the combination of both for 72 hours and analyzed for cellular viability (Fig. 5A). While the cooperative antiproliferative effect was most pronounced in U87 wild-type cells, U87-EGFRvIII and LN229 also revealed an enhanced antiproliferative effect when subjected to the combination treatment compared to treatment with each agent alone (Fig. 5A). We also determined as to whether this enhanced cell death by TRAIL and PJ34 is due to an increase in apoptosis. For that purpose, U87 GBM cells were treated with PJ34, TRAIL or the combination of both for 24 hours and subsequently the amount of specific apoptosis was determined by cell cycle analysis (Fig. 5B). We found that treatment with 20 and 40 µM of PJ34 significantly enhanced TRAIL-mediated cell death/apoptosis when compared to the single agent treatments (Fig. 5B,C). To exclude that the combination treatment of TRAIL and PJ34 requires wild-type p53, we also treated T98G cells with TRAIL, PJ34 and the combination of both reagents for 24 hours and found that in T98G, TRAIL and PJ34 cooperated to induce apoptosis (Figure A in S3 Fig.). Consistent with the degree of cell death activation, (cleavage) of initiator caspase-9 was higher in the combination treatments consisting of TRAIL and PJ34 (20 and 40 µM) (Fig. 5D). Since Olaparib elucidated a marked increase in DR5 levels, we determined the protein levels of DR5 after treatment with PJ34 as well as in combination with the death ligand TRAIL (Fig. 5E). For this purpose, LN229 were treated with 20 µM PJ34 (singly) or in the presence of TRAIL (Fig. 5E). Both conditions led to an up-regulation of DR5 (Fig. 5E). However, only the combination treatment with TRAIL and PJ34 increased activation of caspase-9 and effector caspase-3 (Fig. 5E) - consistent with the enhanced cell death in the combination treatment. In concordance with these findings, combined treatment with TRAIL and PJ34 resulted in an enhanced cleavage of PARP in a dose-dependent manner in U87 and T98G (Figure C in S4 Fig.). Next, we sought to demonstrate that the enhancement of TRAIL-mediated apoptosis by both Olaparib and PJ34 was due to inhibition of PARP-1 and not related to an off-target effect. U87 GBM cells were transfected with a specific siRNA, targeting PARP-1 (Fig. 5F). 72 hours after transfection knock-down of PARP-1 was confirmed by Western Blotting (Fig. 5F). Transfected U87 GBM cells were then treated with increasing concentrations of TRAIL and subjected to analysis for specific apoptosis by flow cytometry (Fig. 5G–H). U87 cells with silenced expression of PARP-1 were significantly more sensitive to the cytotoxic effects of TRAIL, which was accompanied by enhanced activation of effector caspase-3 (Fig. 5F–H).

### The combination of PARP inhibitors with TRAIL is dependent on the proapoptotic protein BAX

U87 cells were treated with TRAIL, PJ34 or the combination of both for 24 hours. Subsequently, cells were harvested, stained with JC-1 to determine the loss of mitochondrial membrane potential after the individual treatments (Figure A–B in S5 Fig.). While PJ34 revealed minor changes in mitochondrial membrane potential, considerable changes were observed in cells treated with TRAIL alone. However, the combination treatment lead to an almost complete dissipation of mitochondrial membrane potential, further confirming the cooperative effects of TRAIL and PARP inhibitors with respect to cell death induction (Figure A–B in S5 Fig.). As the JC-1 stain as well as the activation of caspase-9 suggested a potential involvement of the intrinsic apoptotic pathway, we silenced the expression of BAX, a proapoptotic member of the Bcl-2 family of proteins that is critically involved in the release of cytochrome-c into the cytosol upon intrinsic apoptotic stimulation. U87 cells were transfected with a non-targeting or BAX specific siRNA. 48 hours after transfection cells were treated with the combination of TRAIL and PJ34 for additional 7 hours. Subsequently, cells were harvested and analyzed for the expression of caspase-9, cleaved caspase-3 and Bax (Figure C in S5 Fig.). In the presence of silenced BAX expression, cleavage (activation) of caspases induced by the combination therapy of TRAIL and PJ34 was attenuated, supporting the notion that this treatment regimen requires a mitochondrial amplification loop to maximize its cell death inducing properties. Moreover, U87 cells with silenced Bax expression were treated with the combination of TRAIL and PJ34 for 24 hours and revealed less apoptotic cells (Figure D–E in S5 Fig.).

### TRAIL and PJ34 cooperate to reduce glioma growth *in vivo* and reveal minimal cytotoxicity in non-neoplastic astrocytes

**Figure 6:**
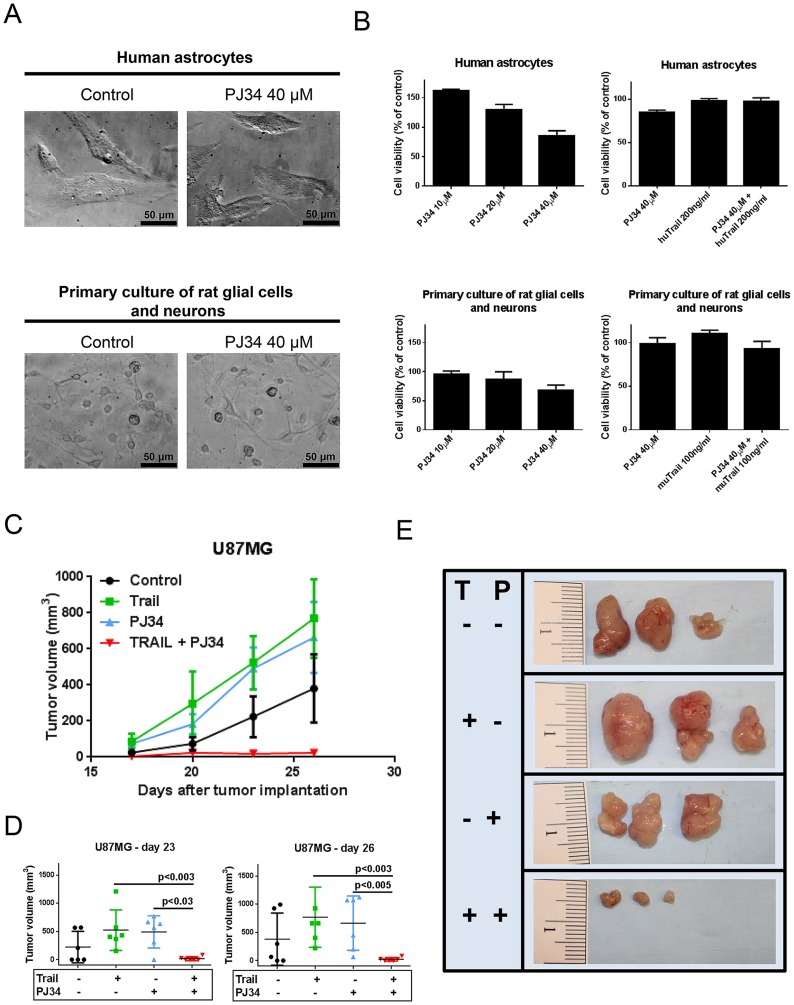
The combination of TRAIL and PJ34 is non-toxic to human astrocytes and primary rat neurons and glial cells and exerts stronger anti-proliferative activity against malignant glioma than the respective single treatments *in vivo*. A) Representative microphotographs of human astrocytes and primary glial/neurons cells treated with PJ34 for 72 hours. B) Human astrocytes and primary neurons/glial cells were treated with increasing concentrations of PJ34 or in combination with human TRAIL (huTRAIL) or murine TRAIL (muTRAIL) for 72 hours and then analyzed by MTT assay. C) Shown is the tumor growth curve with the four different treatment groups: Control (DMSO), TRAIL, PJ34, TRAIL/PJ34 (each n = 6 tumors). Tumors and treatment groups were established as described in the material and methods section. D) Quantification and statistical analysis of treatment groups after 23 and 26 days, respectively. The Mann-Whitney test was used for statistical analysis and a p-value of less than 0.05 was deemed statistically significant. E) Gross images of representative tumors from different treatment groups. T – TRAIL, P – PJ34.

To verify that the combination treatment of PJ34 and TRAIL mainly affects tumor cells this treatment regimen was also tested in non-neoplastic cells. Remarkably, the combination treatment with TRAIL and PJ34 was shown to be non-toxic to normal human astrocytes and primary glial/neuronal cells, suggesting that this treatment will not only be effective against treatment resistant cancers, but also is expected to exert minimal side-effects (Fig. 6A–B). Next, we evaluated whether TRAIL/PJ34 is capable of reducing tumor growth *in vivo*. For that purpose, four different treatment groups (6 tumors in each group) were formed that received treatment with either vehicle, TRAIL, PJ34 or the combination of both as described in the methods section. While the control or single treatments with TRAIL or PJ34 demonstrated a significant growth pattern and increase in tumor size, the tumors in the combination treatment group were significantly smaller (Fig. 6C–E).

## Discussion and Conclusion

Limited therapeutic options are currently available for GBM and high-grade gliomas, which is due to several factors, such as the presence of the blood-brain barrier, the heterogeneity of these tumors [16] and their recalcitrant resistance to apoptosis [17]. Therefore, the quest for novel treatment strategies is on the rise [18], [19]. In the present work, we have provided evidence that glioblastoma cells and tissue widely express PARP-1 and that interfering with PARP-1 might be a suitable strategy to overcome apoptotic resistance. In our experiments, PARP inhibition resulted in an inhibition of proliferation of GBM cells accompanied by a small increase in apoptotic cell death on its own.

TRAIL is a cytokine that has been shown to potently induce apoptosis in neoplastic cells, while leaving most normal cells unaffected. In the last decade, TRAIL has also been studied in clinical trials with limited success thus far [20]. The reasons are multiple, but may include primary or secondary resistance or unfavorable pharmacokinetics [21]. Because TRAIL is a recombinant protein its pharmacokinetics are suboptimal and the molecule is prone to proteolytic degradation. Very recently, a novel molecule was discovered that mediates endogenous induction of TRAIL in tumor cells and thereby triggers their apoptotic suicide [22]. This compound was named TIC10 (TRAIL inducing compound 10) [22]. Having very favorable pharmacokinetics, this molecule crosses the blood brain barrier, induces apoptosis in malignant glioma cells/cultures and cooperates with Bevacizumab to inhibit tumor growth in an orthotopic model of GBM [22]. Mechanistically, TIC10 induced TRAIL at the level of transcription and furthermore even facilitated the expression of DR5 [23] under certain circumstances, sensitizing the cells further to death receptor-mediated apoptosis by TRAIL. Another approach to activate extrinsic apoptosis is by utilizing death receptor-binding antibodies with intrinsic activity. Two representative examples out of this group are mapatumumab and lexatumumab, targeting either DR4 or DR5. These molecules have also reached clinical trials [24]–[27]. Despite the fact that the majority of GBM cells display resistance towards TRAIL, combination treatments were shown to dramatically enhance the killing efficacy of TRAIL. Given the strong up-regulation of DR5 by Olaparib and PJ34 (present study) it was tempting to speculate whether pharmacological or specific siRNA-mediated PARP-1 inhibition could enhance TRAIL-mediated apoptosis in GBM cells. Confirming this notion, pharmacological PARP inhibition sensitized both established GBM cells and low-passage *ex vivo* cultures to the cytotoxic effects of TRAIL *in vitro* as well as in a subcutaneous xenograft model of malignant glioma. In addition, siRNA-mediated specific knock-down of PARP-1 overcame TRAIL resistance in malignant glioma cells accompanied by enhanced activation of caspases. These results are consistent with previous studies showing other DNA-damaging compounds, e.g. Temozolomide, increase expression levels of DR5 and lower the threshold for TRAIL [28]. Specifically, Temozolomide and TRAIL have been combined in a preclinical orthotopic model of GBM and revealed synergistic killing effects *in vivo*. In this study, TRAIL was administered through convection-enhanced delivery (CED), an intratumoral treatment approach being pursued in the context of malignant gliomas. The clear advantages of CED are less systemic side-effects and that higher drug concentrations are achieved within the tumor. Regarding other TRAIL receptors, it appears that DR5 is the main agonistic death receptor in GBM, which is supported by the fact that most gliomas rely on DR5 signaling since a significant proportion of glioma specimens harbor a methylated DR4 promoter and in turn display low to absent mRNA and protein levels [29]. Along these lines, TRAIL-sensitizing reagents that increase the expression of death receptors appear to almost exclusively affect DR5 levels, while leaving DR4 almost unaffected in GBM cells [30]. This fact is also consistent with our present findings in which we do not find a significant change of DR4 levels in response to Olaparib treatment. We also found that PARP-inhibitor mediated up-regulation of DR5 appears to be at least partially dependent on the stress response transcription factor, CCAAT enhancer binding protein (C/EBP) homology protein (CHOP). However, we cannot exclude that potentially other factors are contributing to DR5 up-regulation, such as Erk [31], Sp1 [31], or ATF3 [32], which all three have been described to modulate DR5 levels in response to certain compounds. In addition, we also found that at longer time points and higher concentrations of the PARP-inhibitor CHOP levels declined, which may be due to a feedback mechanism.

Concerning other tumor types it was recently demonstrated that specific PARP-1 knock-down as well as treatment with the PARP inhibitor, PJ34, sensitized pancreatic cancer cells to TRAIL-mediated apoptosis *in vitro* and *in vivo* [33]. These results are generally in agreement with the results presented here, but in contrast Yuan et al. suggested a mechanism of sensitization to TRAIL by PARP-inhibitors that did not involve an increase in TRAIL receptors.

Despite death receptors, TRAIL resistance is determined by a number of other intracellular molecules. For instance, a certain proportion of gliomas reveal low expression of caspase-8 [9], [34] which would be expected to attenuate death receptor-mediated apoptosis. In case of the drug combination of TRAIL with either Olaparib or PJ34 caspase-8 is an instrumental molecule for cell death since down-regulation of caspase-8 strongly suppresses apoptosis induced by the combination therapy. Thus, it appears that for the drug combination of TRAIL with PARP inhibitors the presence of a functional caspase-8 is a prerequisite. Furthermore, c-FLIP is an endogenous inhibitor of caspase-8 and has been associated with TRAIL-resistance in cancers. C-FLIP consists of at least three splicing variants, a more recently described c-FLIP (R) form, a long form (c-FLIP (L) and a short form (c-FLIP (S)) [35]. Each of which have roles in regulating extrinsic apoptosis. Depending on the tumor type and drug combination treatment either one of the forms appears to be more important in apoptosis inhibition. Pharmacological modulation of c-FLIP levels were achieved by several compounds including histone-deacetylase inhibitors, mitochondrial Hsp90 inhibitors [12], proteasomal inhibitors [36], flavonoids [37] and chemotherapeutics [38] among others. With respect to histone-deacetylase inhibitors, it was recently found that these drugs modulate c-FLIP levels at the level of transcription through c-myc, which suppresses c-FLIP transcription [39]. In the present study, PARP inhibitors did not modulate the caspase-8 levels. Other factors that modulate TRAIL-resistance are the Inhibitor of Apoptosis Proteins (IAPs) [40], [41], XIAP and survivin and the anti-apoptotic Bcl-2 family of proteins, such as Bcl-2, Bcl-xL and Mcl-1. In this context, down-regulation of survivin by flavonoids has been shown to enhance TRAIL-mediated apoptosis in GBM cells. Similarly, inhibition of Bcl-xL and Bcl-2 by ABT-737 is known to drive death receptor-mediated apoptosis in malignant glioma and other tumor entities [42]. Elucidating additional off-target effects, ABT-737 was also shown to increase death receptor expression in cancer cells [43], thereby further facilitating its sensitizing effects for death ligands even at the level of the death-inducing-signaling-complex (DISC-complex).

In summary, we have provided a framework for a novel treatment regimen for malignant glioma that mechanistically relies on the reactivation of extrinsic apoptotic cell signaling by induction of death receptor expression. This treatment is active *in vivo* and also effective against stem cell-like glioma cells, a specific cellular fraction of tumor cells that drive recurrence and treatment resistance.

## Supporting Information

S1 Fig **Expression levels of PARP-1 in GBM cells and GBM tissue microarrays (TMAs).** A) GBM tissue microarrays (TMAs), containing 34 tumor samples, were stained with an antibody against PARP-1. Representative micro photographs were taken from two GBMs and one representative sample of adjacent normal brain tissue. B) Cell lysates were prepared from three established GBM cell lines, U87, LN229, and U373 cells as well as from three neurosphere glioma cell cultures, NCH644, GS9-6 and NCH690. PARP-1 protein expression was analyzed by immunoblotting. One star “*” indicates short term exposure, whereas two stars “**” show a longer exposure for the same immunoblot of PARP-1. C) Chemical structure of the PARP inhibitor, Olaparib. D) LN229 GBM cells were treated with Olaparib (10 µM) for 72 hours and subjected to cell cycle analysis by flow cytometry. sG1 – sub G1 fraction (apoptotic cell fraction). E) U87, U87-EGFRvIII, LN229 GBM cells and GS9-6 GBM neurosphere culture were treated with increasing concentrations of the PARP inhibitor, Olaparib, and after 72 hours subjected to analysis of cellular viability by MTT assay. Values are provided as mean ± SEM of replicates of a representative experiment.
(TIF)

S2 Fig **Inhibition of components of the DISC–complex interferes with engagement of apoptosis induced by TRAIL/PARP inhibitors. Requirements of TRAIL/Olaparib mediated cell death.** A) U87 GBM cells were transfected with a non-targeting siRNA or a caspase-8-specific siRNA. 72 hours after transfection cells were treated with the combination of TRAIL (100 ng/ml) and Olaparib (10 µM) for 7 hours, harvested for immunoblotting and analyzed for expression of full length caspase-8 (FL-CP8) and cleaved caspase-3 (cCP3). B) U87 cells were transfected as in (A). Subsequently cells were treated with the combination of TRAIL (100 ng/ml) and Olaparib (10 µM) for 24 hours, harvested and analyzed for the amount of apoptotic cells (sub-G1 fraction) by flow cytometry. C) LN229 GBM cells were transfected with a non-targeting siRNA or a caspase-8-specific siRNA. 72 hours after transfection cells were treated with the combination of TRAIL (200 ng/ml) and Olaparib (10 µM) for 7 hours, harvested for immunoblotting and analyzed for expression of full length caspase-8 (FL-CP8) and cleaved caspase-3 (cCP3). D) LN229 cells were transfected as in (C). Subsequently cells were treated with TRAIL (200 ng/ml) and Olaparib (10 µM) for 24 hours, harvested and analyzed for the amount of apoptotic cells (sub-G1 fraction) by flow cytometry. E) U87 cells were transfected with a non-targeting or a caspase-8-specific siRNA and subsequently treated with the combination of TRAIL and PJ34. Cells were analyzed for specific apoptosis and representative plots are provided. F) U87 cells were transfected with a DR5-specific siRNA for 48 hours, treated with the combination of TRAIL/Olaparib for 7 hours and analyzed for the expression of DR5 and cleavage of caspase-3 by immunoblotting. TR – TRAIL, Olap – Olaparib.
(TIF)

S3 Fig **Apoptosis induction by TRAIL/PJ34 in T98G (** ***TP53*** **mutated) glioblastoma cells.** A) T98G cells were treated with TRAIL (200 ng/ml), PJ34 (40 µM) or the combination of both and analyzed for apoptosis by staining for Propidium iodide and subsequent flow cytometry.
(TIF)

S4 Fig **U251 and GS9-6 glioblastoma cells were stained with annexin V and PI prior to flowcytometric analysis.** A,B): Quantitative representation of the fraction of annexin V-positive/PI-negative cells (early apoptosis), the fraction of annexin V-negative/PI-positive cells (necrosis) and the fraction of annexin V-positive/PI-positive cells (late apoptosis/necrosis) in U251 (A) and GS9-6 (B) glioblastoma cells subjected to treatment with olaparib (olap), Trail (TR) the combination of both compounds or solvent for 24 hours. C) U87 and T98G cells were treated with TRAIL (100 ng/ml), indicated concentrations of PJ34 or the respective combinations of both agents for 7 hours prior to harvesting for immunoblotting and analysis for expression of PARP-1 and cleaved PARP.
(TIF)

S5 Fig **Requirement of mitochondrial amplification for the combination therapy of TRAIL/PJ34.** A-B) U87 cells were treated with TRAIL (100 ng/ml), PJ34 (40 µM) or the combination of both for 24 hours. Subsequently, cells were harvested, stained with JC-1 and analyzed for loss of mitochondrial membrane potential by flow cytometry (red channel - FL2-H). A) Representative histograms after staining for JC-1. B) Quantitative representation of the results for the JC-1 staining. C) U87 GBM cells were transfected with a non-targeting or a BAX-specific siRNA. 48 hours after transfection cells were subjected to treatment with TRAIL (100 ng/ml) and PJ34 (20 µM) for 7 hours and harvested for immunoblotting to determine protein levels of caspase-9 (CP9), cleaved caspase-3 (cCP3) and BAX. D–E) In addition, BAX siRNA transfected cells were treated as above for 24 hours. Following the incubation, cells were harvested for analysis for apoptosis by flow cytometry. Representative plots and a quantitation of the results are provided in D and E, respectively. Columns, mean; bars, SEM. TR – TRAIL.
(TIF)

S1 Table **Expression levels of PARP-1 in GBM tissue specimens.**
(DOC)

S1 File **Additional Methods.**
(DOC)
