# Supplementary figures and images for: PARP Inhibition Restores Extrinsic Apoptotic Sensitivity in Glioblastoma
Source: PLoS One. 2014 Dec 22;9(12):e114583. doi: 10.1371/journal.pone.0114583 (PMC4273972; doi:10.1371/journal.pone.0114583)

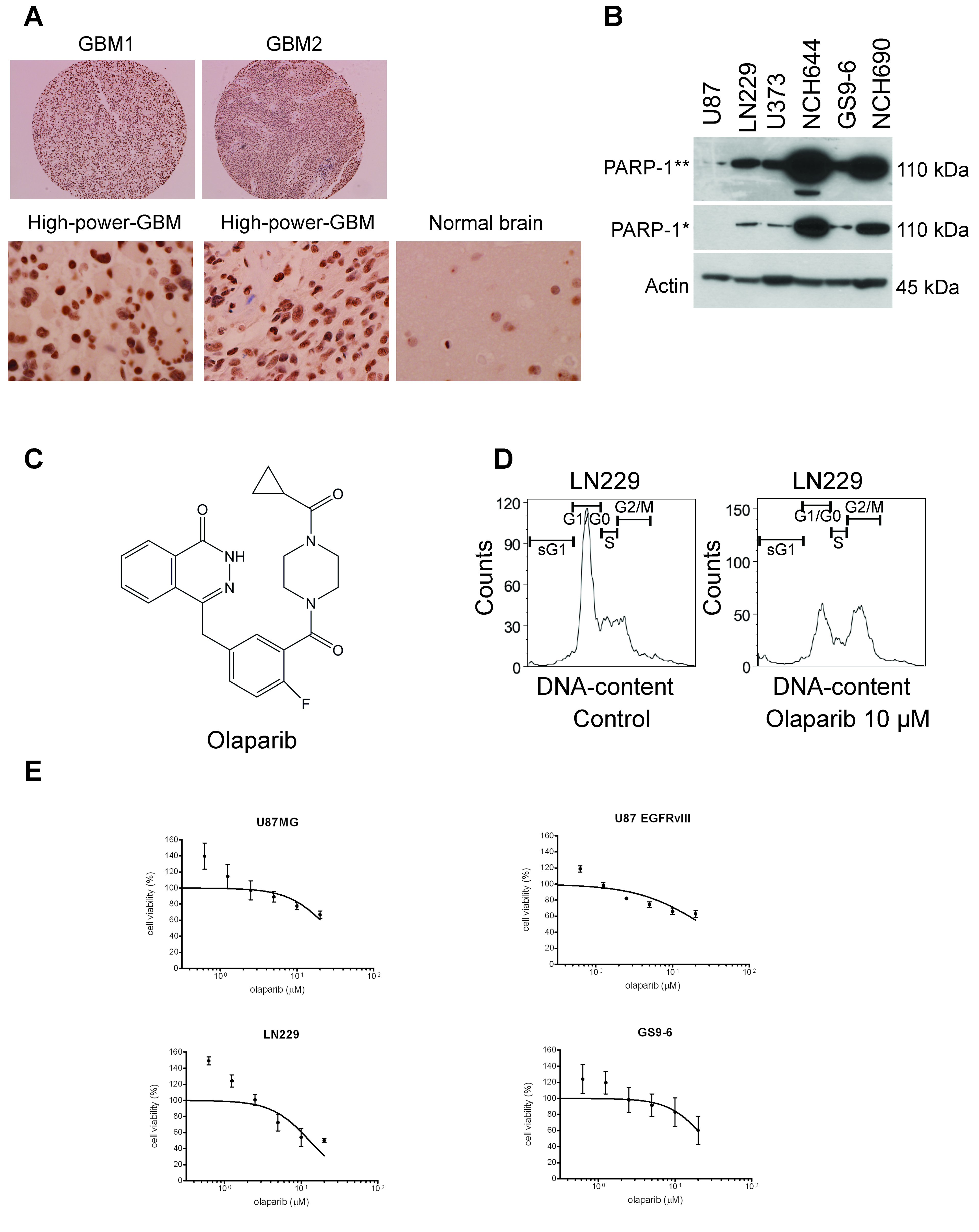

Supplement: S1 Fig — Expression levels of PARP-1 in GBM cells and GBM tissue microarrays (TMAs). A) GBM tissue microarrays (TMAs), containing 34 tumor samples, were stained with an antibody against PARP-1. Representative micro photographs were taken from two GBMs and one representative sample of adjacent normal brain tissue. B) Cell lysates were prepared from three established GBM cell lines, U87, LN229, and U373 cells as well as from three neurosphere glioma cell cultures, NCH644, GS9-6 and NCH690. PARP-1 protein expression was analyzed by immunoblotting. One star “*” indicates short term exposure, whereas two stars “**” show a longer exposure for the same immunoblot of PARP-1. C) Chemical structure of the PARP inhibitor, Olaparib. D) LN229 GBM cells were treated with Olaparib (10 µM) for 72 hours and subjected to cell cycle analysis by flow cytometry. sG1 – sub G1 fraction (apoptotic cell fraction). E) U87, U87-EGFRvIII, LN229 GBM cells and GS9-6 GBM neurosphere culture were treated with increasing concentrations of the PARP inhibitor, Olaparib, and after 72 hours subjected to analysis of cellular viability by MTT assay. Values are provided as mean ± SEM of replicates of a representative experiment. (TIF) [file pone.0114583.s001.tif]

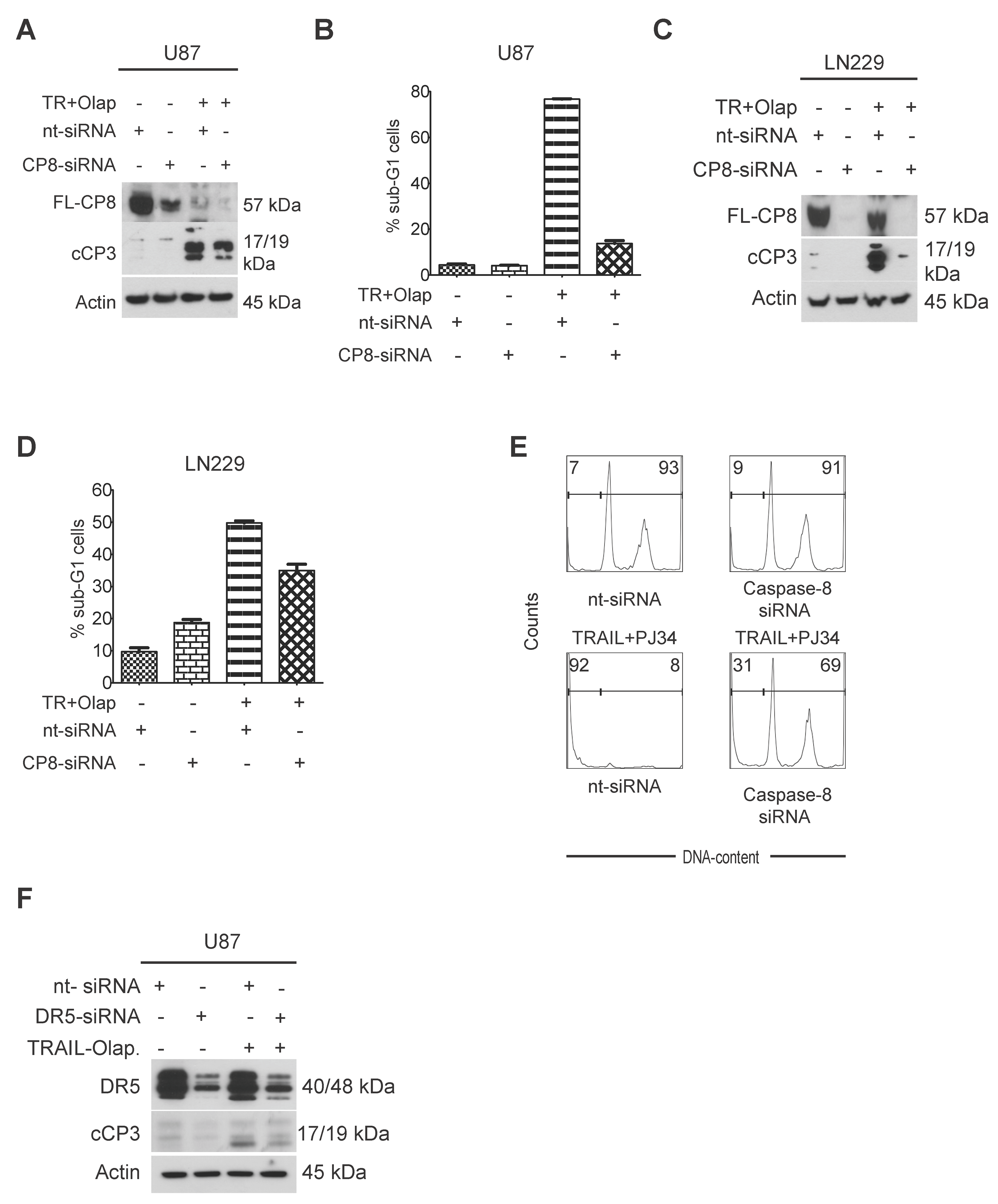

Supplement: S2 Fig — Inhibition of components of the DISC–complex interferes with engagement of apoptosis induced by TRAIL/PARP inhibitors. Requirements of TRAIL/Olaparib mediated cell death. A) U87 GBM cells were transfected with a non-targeting siRNA or a caspase-8-specific siRNA. 72 hours after transfection cells were treated with the combination of TRAIL (100 ng/ml) and Olaparib (10 µM) for 7 hours, harvested for immunoblotting and analyzed for expression of full length caspase-8 (FL-CP8) and cleaved caspase-3 (cCP3). B) U87 cells were transfected as in (A). Subsequently cells were treated with the combination of TRAIL (100 ng/ml) and Olaparib (10 µM) for 24 hours, harvested and analyzed for the amount of apoptotic cells (sub-G1 fraction) by flow cytometry. C) LN229 GBM cells were transfected with a non-targeting siRNA or a caspase-8-specific siRNA. 72 hours after transfection cells were treated with the combination of TRAIL (200 ng/ml) and Olaparib (10 µM) for 7 hours, harvested for immunoblotting and analyzed for expression of full length caspase-8 (FL-CP8) and cleaved caspase-3 (cCP3). D) LN229 cells were transfected as in (C). Subsequently cells were treated with TRAIL (200 ng/ml) and Olaparib (10 µM) for 24 hours, harvested and analyzed for the amount of apoptotic cells (sub-G1 fraction) by flow cytometry. E) U87 cells were transfected with a non-targeting or a caspase-8-specific siRNA and subsequently treated with the combination of TRAIL and PJ34. Cells were analyzed for specific apoptosis and representative plots are provided. F) U87 cells were transfected with a DR5-specific siRNA for 48 hours, treated with the combination of TRAIL/Olaparib for 7 hours and analyzed for the expression of DR5 and cleavage of caspase-3 by immunoblotting. TR – TRAIL, Olap – Olaparib. (TIF) [file pone.0114583.s002.tif]

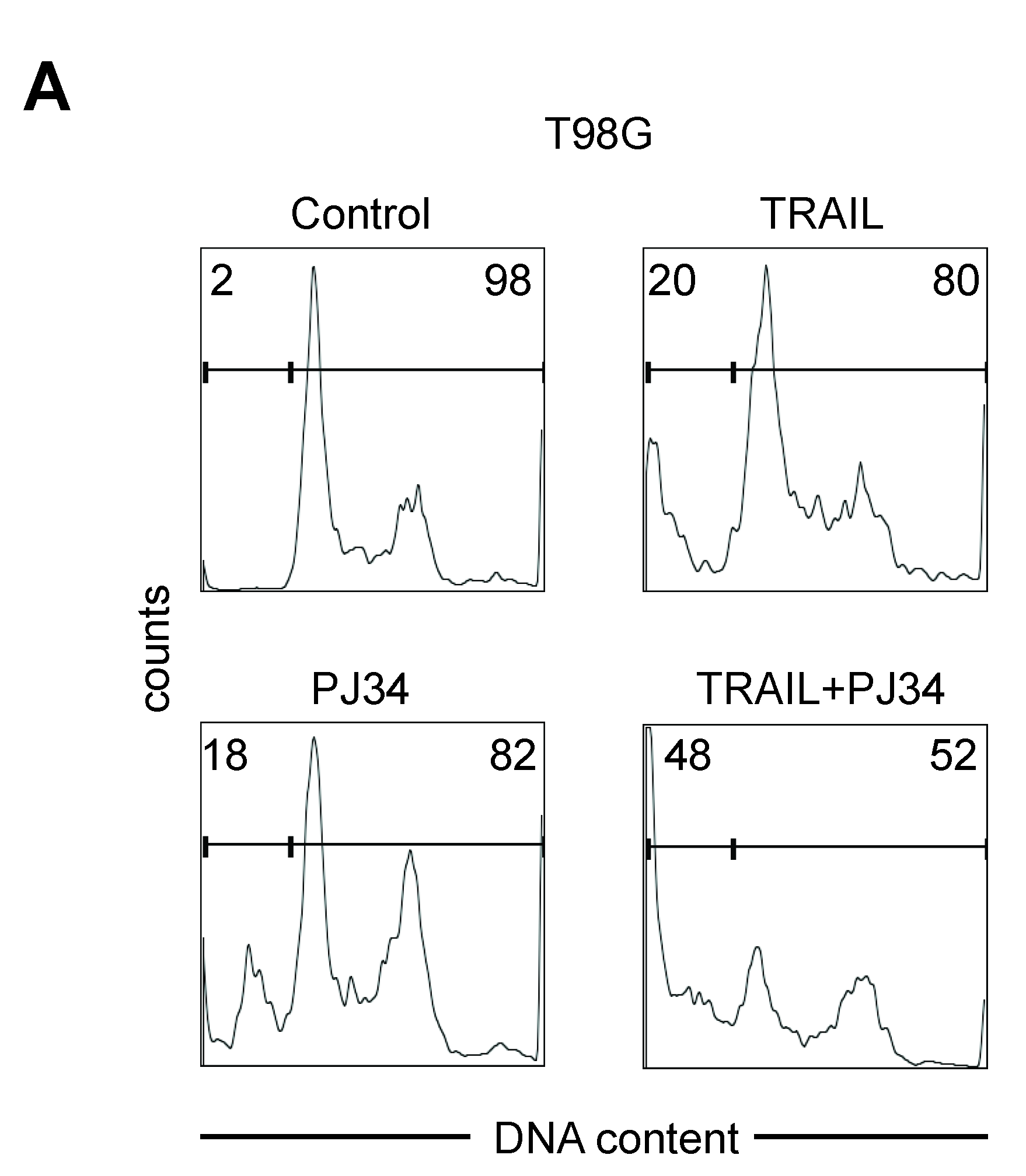

Supplement: S3 Fig — Apoptosis induction by TRAIL/PJ34 in T98G ( TP53 mutated) glioblastoma cells. A) T98G cells were treated with TRAIL (200 ng/ml), PJ34 (40 µM) or the combination of both and analyzed for apoptosis by staining for Propidium iodide and subsequent flow cytometry. (TIF) [file pone.0114583.s003.tif]

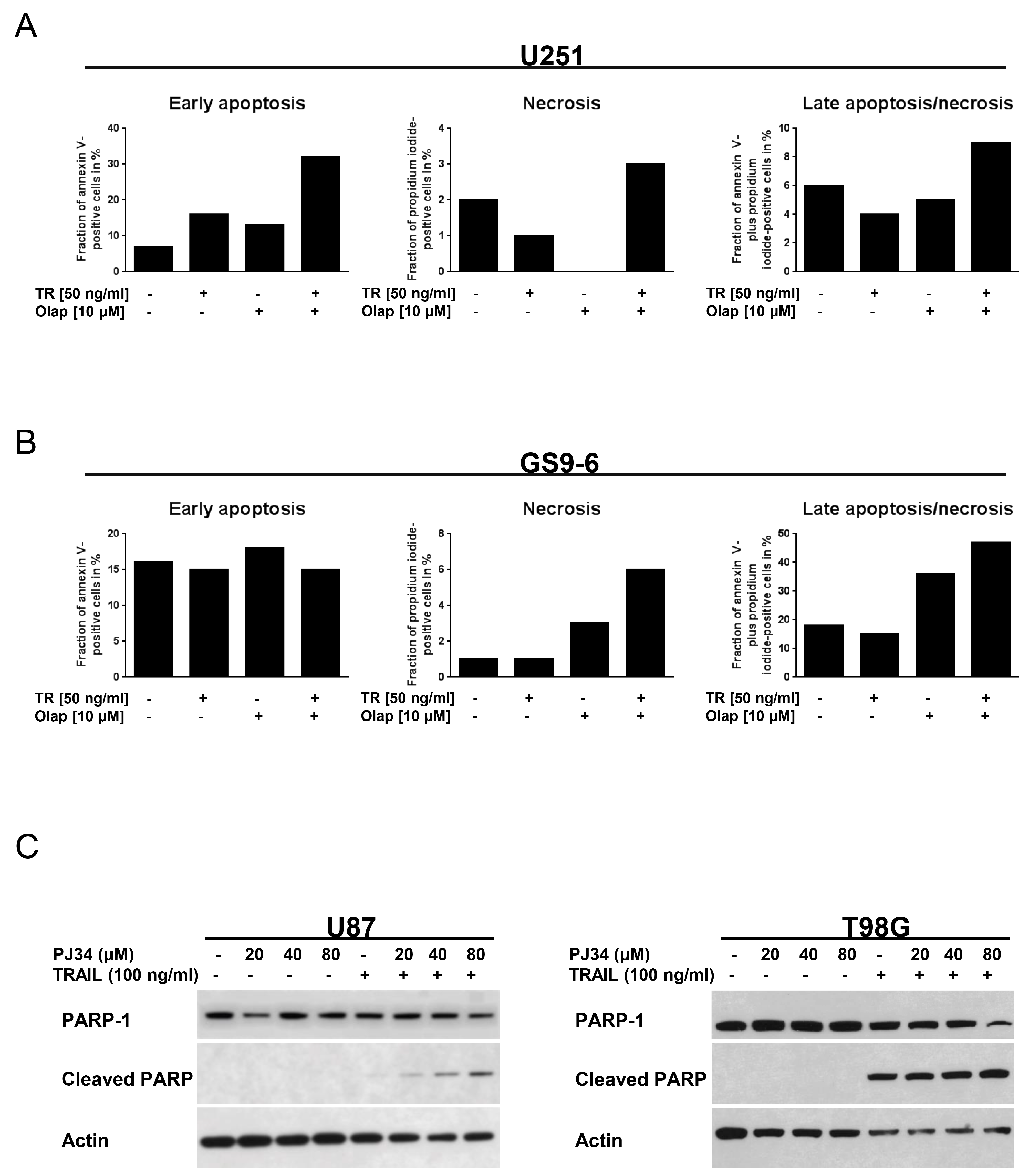

Supplement: S4 Fig — U251 and GS9-6 glioblastoma cells were stained with annexin V and PI prior to flowcytometric analysis. A,B): Quantitative representation of the fraction of annexin V-positive/PI-negative cells (early apoptosis), the fraction of annexin V-negative/PI-positive cells (necrosis) and the fraction of annexin V-positive/PI-positive cells (late apoptosis/necrosis) in U251 (A) and GS9-6 (B) glioblastoma cells subjected to treatment with olaparib (olap), Trail (TR) the combination of both compounds or solvent for 24 hours. C) U87 and T98G cells were treated with TRAIL (100 ng/ml), indicated concentrations of PJ34 or the respective combinations of both agents for 7 hours prior to harvesting for immunoblotting and analysis for expression of PARP-1 and cleaved PARP. (TIF) [file pone.0114583.s004.tif]

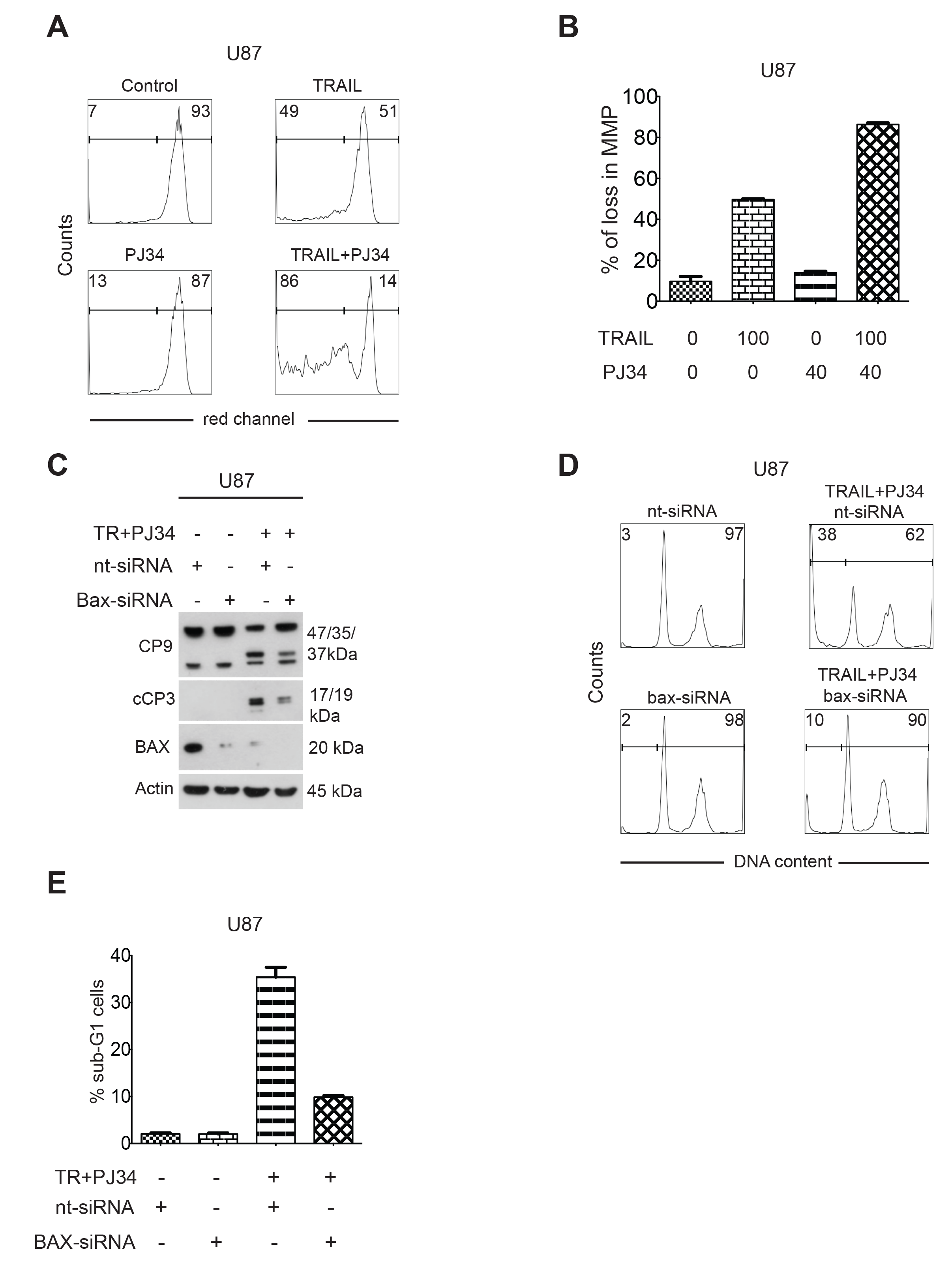

Supplement: S5 Fig — Requirement of mitochondrial amplification for the combination therapy of TRAIL/PJ34. A-B) U87 cells were treated with TRAIL (100 ng/ml), PJ34 (40 µM) or the combination of both for 24 hours. Subsequently, cells were harvested, stained with JC-1 and analyzed for loss of mitochondrial membrane potential by flow cytometry (red channel - FL2-H). A) Representative histograms after staining for JC-1. B) Quantitative representation of the results for the JC-1 staining. C) U87 GBM cells were transfected with a non-targeting or a BAX-specific siRNA. 48 hours after transfection cells were subjected to treatment with TRAIL (100 ng/ml) and PJ34 (20 µM) for 7 hours and harvested for immunoblotting to determine protein levels of caspase-9 (CP9), cleaved caspase-3 (cCP3) and BAX. D–E) In addition, BAX siRNA transfected cells were treated as above for 24 hours. Following the incubation, cells were harvested for analysis for apoptosis by flow cytometry. Representative plots and a quantitation of the results are provided in D and E, respectively. Columns, mean; bars, SEM. TR – TRAIL. (TIF) [file pone.0114583.s005.tif]
