# Supplementary material for: PARP Inhibition Restores Extrinsic Apoptotic Sensitivity in Glioblastoma
Source: PLoS One. 2014 Dec 22;9(12):e114583. doi: 10.1371/journal.pone.0114583 (PMC4273972; doi:10.1371/journal.pone.0114583)
Supplement: S1 Table — Expression levels of PARP-1 in GBM tissue specimens. (DOC) [file pone.0114583.s006.doc]

**Table S1:**

| **Table 1.** Expression levels of PARP-1 in GBM tissue specimens. | | |
| --- | --- | --- |
| Protein | Score | Tumors (%) |
| PARP-1 | 0 | 0/34 (0%) |
|  | 1 | 23/34 (68%) |
|  | 2 | 11/34 (32%) |
| 0 = no staining, 1 = low-moderate expression, 2 = high expression | | |
