# Supplementary material for: PARP Inhibition Restores Extrinsic Apoptotic Sensitivity in Glioblastoma
Source: PLoS One. 2014 Dec 22;9(12):e114583. doi: 10.1371/journal.pone.0114583 (PMC4273972; doi:10.1371/journal.pone.0114583)
Supplement: S1 File — Additional Methods. (DOC) [file pone.0114583.s007.doc]

**Supporting Information S1. Additional Methods:**

Immunohistochemistry of GBM specimens

Tissue microarrays (TMAs), containing 34 de-identified GBM specimens (in triplicate), were provided by the Division of Neuropathology at the Columbia University Medical Center. TMAs were created by removing three one-millimeter cores of GBM tumors. Immunohistochemical staining was performed as previously described (1). Briefly, 4 μM thick sections were cut and deparaffinized in xylene. For antigen retrieval sections were pretreated by boiling samples in 0.01M citrate buffer (pH 6.0) for 5min. TMAs were stained with a dilution of 1:100 of anti-PARP-1 (F-2, #sc-8007, Santa Cruz Biotechnology Inc.). The secondary antibodies included anti-mouse polymeric antibodies (purchased from Dako) and diaminobenzidine was used as chromogene. TMAs were scored, using a 3-tier scoring system (0: no expression, 1: weak expression (weak to moderate nuclear staining in tumor cells, 2: strong expression (majority of tumor cells with strong nuclear expression). A final score for each tumor was reached based on the highest scoring core (out of three cores per GBM specimen). Representative microphotographs of the TMAs were taken.

Rat mixed glial/neuronal primary culture

Mixed postnatal primary rat brain cultures were prepared as previously described (2, 3). Briefly, the rostral part of the cerebral cortex of 2-day-old rat pups was harvested and minced into 1 mm^3^ sized pieces in ice-cold phosphate-buffered saline. The pieces of tissue were enzymatically dissociated with 20 U/ml papain in 1 mM cysteine, 0.001% phenol red, 116 mM NaCl, 5.4 mM KO, 26 mM NaHCO3, 2 mM NaH2PO4, 1 mM magnesium phosphate, 500 µM EDTA, and 25 mM glucose, pH 7.3 for 2 h. Then, the pieces of tissue were washed 3 times with medium containing 90% minimal essential medium, 10% calf serum, 1.83 mM glucose, 5 µg/ml bovine pancreatic insulin, 500 µM glutamine, 0.6 U/mI penicillin, and 60 µg/ml streptomycin (M10C-G) and triturated 3-5 times. Afterwards, cells were pelleted and resuspended in M10C-G medium prior to being plated at a density of 100,000 cells/well on 96-well plates which were precoated with poly-d-lysine and 10µg/ml laminin. After 1 h of incubation at 37°C, the cells were washed once with Eagle’s minimal essential medium prior to adding 100µl/well of M10C-G medium.

Analysis of membranous DR5 expression

U87 GBM cells were stained with an isotype control, Mouse IgG2B Phycoerythrin Isotype Control (Clone 133303), or an antibody against DR5, Human TRAIL R2/TNFRSF10B Phycoerythrin MAb (Clone 71908) (Both from R&D Systems, Minneapolis, MN) according to the manufacturer’s instructions. Briefly, 1x10^5^ cells were washed three times with PBS and resuspended in 500 μl of PBS prior to adding 10 μl of the DR5 antibody or isotype control and incubation for 30 min at 4ºC. Then, cells were washed twice with PBS, resuspended in 400 μl of PBS and subjected to flow cytometric analysis on a FACS Calibur machine (Becton Dickinson).

**Supplementary References:**

1. Monaco SE, Angelastro JM, Szabolcs M, Greene LA. The transcription factor ATF5 is widely expressed in carcinomas, and interference with its function selectively kills neoplastic, but not nontransformed, breast cell lines. International journal of cancer Journal international du cancer. 2007;120(9):1883-90. PubMed PMID: 17266024.

2. Mena MA, Davila V, Sulzer D. Neurotrophic effects of L-DOPA in postnatal midbrain dopamine neuron/cortical astrocyte cocultures. Journal of neurochemistry. 1997;69(4):1398-408. PubMed PMID: 9326268.

3. Rayport S, Sulzer D, Shi WX, Sawasdikosol S, Monaco J, Batson D, Rajendran G. Identified postnatal mesolimbic dopamine neurons in culture: morphology and electrophysiology. The Journal of neuroscience : the official journal of the Society for Neuroscience. 1992;12(11):4264-80. PubMed PMID: 1359033.
